# Visit-Associated Logistics Framework

**DOI:** 10.1093/geroni/igaf122.909

**Published:** 2025-12-31

**Authors:** Lauren Moo, Victoria Ngo, Elizabeth Marfeo, Steven Shirk, Elizabeth Chamberlin

**Affiliations:** VA Bedford Healthcare System, Bedford, Massachusetts, United States; VA Bedford Health Care System, Bedford, Massachusetts, United States; Tufts University, Medford, Massachusetts, United States; VA Bedford Health Care System, Bedford, Massachusetts, United States; VA Bedford Health Care System, Bedford, Massachusetts, United States

## Abstract

Coordinating in-person clinical visits requires complex logistical planning for the family caregivers of older adults. We developed a conceptual framework integrating concepts from geriatric research and visit logistics that conceptualizes how visit-related challenges can impact caregiving and attendance of medical appointments. In this session, we will introduce the Visit-Associated Logistics (VAL) Framework that includes domains of Scheduling, Preparing for the visit, Visit-related time and travel, Caregiver independence, Routine disruption, and Caregiver feelings and impressions.

